# Effect of Long-Term Sodium Salicylate Administration on Learning, Memory, and Neurogenesis in the Rat Hippocampus

**DOI:** 10.1155/2018/7807426

**Published:** 2018-04-01

**Authors:** Haichen Niu, Sheng Ding, Haiying Li, Jianfeng Wei, Chao Ren, Xiujuan Wu, Tanzeel Huma, Qiang Zhang

**Affiliations:** ^1^Department of Genetics, Xuzhou Medical University, Xuzhou, Jiangsu 221006, China; ^2^School of Public Health, Xuzhou Medical University, Xuzhou, Jiangsu 221006, China; ^3^Department of Pathology, Xuzhou Medical University, Xuzhou, Jiangsu 221006, China; ^4^Department of Histology and Embryology, School of Basic Medical Sciences, Xuzhou Medical University, Xuzhou, Jiangsu 221006, China; ^5^Department of Neurology, The Affiliated Yantai Yuhuangding Hospital of Qingdao University, Yantai, Shandong 264000, China; ^6^Institute of Molecular Biology and Biotechnology, University of Lahore, Lahore 54000, Pakistan

## Abstract

Tinnitus is thought to be caused by damage to the auditory and nonauditory system due to exposure to loud noise, aging, or other etiologies. However, at present, the exact neurophysiological basis of chronic tinnitus remains unknown. To explore whether the function of the limbic system is disturbed in tinnitus, the hippocampus was selected, which plays a vital role in learning and memory. The hippocampal function was examined with a learning and memory procedure. For this purpose, sodium salicylate (NaSal) was used to create a rat animal model of tinnitus, evaluated with prepulse inhibition behavior (PPI). The acquisition and retrieval abilities of spatial memory were measured using the Morris water maze (MWM) in NaSal-treated and control animals, followed by observation of c-Fos and delta-FosB protein expression in the hippocampal field by immunohistochemistry. To further identify the neural substrate for memory change in tinnitus, neurogenesis in the subgranular zone of the dentate gyrus (DG) was compared between the NaSal group and the control group. The results showed that acquisition and retrieval of spatial memory were impaired by NaSal treatment. The expression of c-Fos and delta-FosB protein was also inhibited in NaSal-treated animals. Simultaneously, neurogenesis in the DG was also impaired in tinnitus animals. In general, our data suggest that the hippocampal system (limbic system) may play a key role in tinnitus pathology.

## 1. Introduction

Tinnitus is a conscious awareness of sound without an external source of the sound. It is described by a patient as a ringing or buzzing in one or both ears in the absence of an auditory stimulus. The prevalence rate of chronic tinnitus ranges from approximately 5% to 15% of the population [1]. Studies indicate that any level of the auditory pathway, including the cochlear nerve, acoustic nerve, or central auditory pathways, can induce tinnitus [2]. Tinnitus can induce many other symptoms including anxiety, emotional disorders, sleep disturbance, and work impairment [3]. Tinnitus affects the quality of life in general [4]. However, a number of neuroimaging studies in humans indicate that the limbic system may have functional and anatomical roles in the tinnitus-related field, outside of the central auditory pathways. In this disorder, limbic changes were the result of tinnitus, not the cause. It seems that central auditory dysfunction may not be the only target for understanding chronic tinnitus.

The hippocampus, which is a part of the limbic system, plays a vital role in learning and memory process. Sensory systems can transduce information of the external stimulus to neural representations in the central nervous system (CNS), which will help the memory system to establish a memory trace [5]. Accurately, the auditory system can transfer external information to the memory system by transforming it into memory-related information. For long-term auditory memory, auditory information is first processed in the hippocampus to form a short-term memory. Later, the short-term memory is transformed into a long-term memory [6]. Preliminary studies in primates indicated that large medial temporal lobe lesions impair auditory recognition memory [7]. However, at present, there is no reference study indicating the effect on nonauditory memory. It is unclear whether the nonauditory memory of tinnitus is affected or not.

In the hippocampus, the subgranular zone of the dentate gyrus (DG) continually generates new neurons in adulthood. These new neurons synaptically integrate into hippocampal networks and provide potential substrates for learning. Continuous integration of new neurons affects the establishment of memories at the circuit level of the hippocampus. New neurons integrate into the hippocampus by competing with existing cells in the neural memory network, establishing new memory circuits that may coexist with or even replace first memory circuits. In addition, neurogenesis can be impaired by pathological changes or pharmacological treatments, resulting in disturbance of learning and memory.

Sodium salicylate (NaSal) is frequently used in clinical settings and has the potential to cause reversible tinnitus and sensorineural hearing loss [8]. NaSal-induced tinnitus in rats is a popular animal model for the study of tinnitus and has been used in many substantial pharmacological and pathological studies [8, 9].

Previous studies suggested that tinnitus originated at the cochlear level and developed in the CNS, resulting in neuroplasticity-related dysfunction. At the same time, many studies indicated that hippocampal plasticity promotes the learning and memory procedure in rodents and humans. But so far, it is unknown whether NaSal treatment will have effects on the learning and memory procedure. We designed this study to investigate whether tinnitus affects the hippocampal function or not. For this purpose, we developed a tinnitus rat model by inducing tinnitus in rats via long-term NaSal administration, and tinnitus-like behavior was confirmed using the prepulse inhibition (PPI) test. The Morris water maze (MWM) was used to examine the nonauditory learning ability in tinnitus rats, and neurogenesis of the DG was observed using an immunohistochemical tool. The results of this study will help to elucidate the contribution to learning and memory in the hippocampus during tinnitus induced by sodium salicylate.

## 2. Materials and Methods

### 2.1. Animals

In this study, male Sprague-Dawley rats were used (200–220 g). Rats were kept on a 12/12 h dark/light cycle with the light phase starting at 7 p.m., with ad libitum access to food and water. Experimental procedures were in accordance with the guidelines and regulations set out by Xuzhou Medical University. All experiments were approved by the Institutional Animal Care and Use of Xuzhou Medical University under the animal protocol number 2015A1204B07. Rats were subjected to intraperitoneal (i.p.) injection of sodium salicylate 300 mg/kg (Sigma, S3007, Shanghai, China) daily for 7 or 14 consecutive days. The control groups were treated with saline for 7 or 14 consecutive days. Behavior tests were performed 2 h after the last injection, as previously described [10].

### 2.2. Tinnitus Tests

To test the auditory stimulus-specific gap detection deficits, we developed a custom-made auditory setup to induce prepulse inhibition (PPI) in the animals. Tests were performed in a sound-attenuating chamber. The PPI procedure was done as previously described [11, 12]. For the gap detection tests that were used to evaluate tinnitus perception, all animals were located in a sound-attenuating room with a 60 dB ambient noise level. During every experiment, animals were habituated to the device fixed above a platform to a gravity accelerometer and were exposed to 65 dB of sound pressure level (SPL) white background noise. This signal was detected by a sensitive sensor and transferred to a computer in an adjacent room to collect the startle-response data. During the test session, the rats were first presented with ten trials of randomly delivered 115 dB SPL pulses. Then, animals were given 30 trials of randomly delivered acoustic stimuli delivered through a speaker. The acoustic stimuli consisted of 10 trials of a 115 dB SPL pulse stimulus, 10 trials without a delivered stimulus (NOSTIM), and 30 trials of a prepulse startle stimulus. The SPL pulse was a single 20 ms sound presentation. The prepulse startle stimuli were 100 ms of 20 ms white-noise pulses that contained nonstartling stimuli of 75, 80, or 85 dB SPL (PPI2, PPI4, and PPI8, resp.) followed by a single 20 ms 115 dB SPL pulse. Intertrial intervals (ITIs) of 27–32 s were used between the stimuli presentations. PPI was quantified as the percent decrease in the peak amplitude of the startle response (ASR) when a prepulse preceded the startling noise in comparison to the amplitude when no gap or prepulse was present [(1 − PPI/ASR) × 100].

### 2.3. Spatial Memory Tests

The MWM was used to evaluate the spatial memory abilities in rats [13], which consisted of a circular plastic pool filled with warm water with the addition of nontoxic yellow paint. A video camera was suspended above the pool to track the animals which were connected to a computer with a tracing system. Different visual cues were placed on the surrounding walls so that the rats could use them for spatial orientation and remained unchanged during the experimental period.

24 hours after tinnitus examination rats were trained for four days in a water maze by doing four trials per day. On the 4th day, the latency to find the hidden platform underwater was also recorded. A maximum time of 90 s was provided to each experimental rat. Each trial had a different starting point, and if they failed to find the platform in 90 s, the rats would be guided to the platform manually and kept on the platform for 10 s. The time taken by each rat to reach the platform (latency) was recorded. The spatial probe test was conducted on the fifth day. This test is used to evaluate reversal learning, revealing whether or not animals can extinguish their initial learning of the platform's position and acquire a direct path to the new goal position. The platform was removed, and each rat was released opposite the target quadrant (the southeast quadrant), facing the wall of the pool. In the probe test, the time that the rats spent getting to the target quadrant was recorded to assess their spatial memory ability.

### 2.4. c-Fos and delta-FosB Immunohistochemistry (IHC)

For c-Fos IHC experiments, animals were anesthetized with trichloroacetaldehyde monohydrate (chloral hydrate; 10%, 0.3 ml/100 g, Sinopharm Chemical Reagent Co., Ltd., Shanghai, China) 4 h after acquisition training on the 4th day, and for delta-FosB IHC experiment, animals were anesthetized with chloral hydrate 48 h after a second probe test (7th day). Animals were then immediately perfused transcardially with 100 ml of 0.9% saline in 0.01 M (pH = 7.2) phosphate-buffered saline (PBS), followed by 200 ml of 4% paraformaldehyde (PFA) in 0.01 M PBS (pH = 7.2). Brains were extracted followed by fixation in 4% PFA and stored overnight at 4°C. After 24 h, the brains were dehydrated by 10%, 20%, and 30% sugar in 0.01 M PBS (pH = 7.2) until the brains sunk to the bottom at 4°C. Then, consecutive coronal sections (thickness = 25 *μ*m) of hippocampal fields were cut using Leica freezing microtome (Leica, CM1950).

For immunostaining in the hippocampus, three slices per animal per target brain field were selected. The selected brain slices were washed three times for 3 min in PBS and then washed in a buffer containing 5% normal goat serum and 0.5% Triton X-100 in PBS for 30 min. Later, these slices were incubated with polyclonal goat anti-c-Fos 1 : 200 (sc-52; Santa Cruz Biotechnology, Shanghai, China) or anti-ΔFosB (H-75, sc-48, Santa Cruz Biotechnology, Shanghai, China) overnight at 4°C. Sections were rinsed three times for 3 min in 0.01 M PBS and incubated for 1 h at room temperature with the appropriate secondary antibodies. After washing, sections were incubated with the avidin-biotin-peroxidase complex for 1 h (ABC kit, Beyotime Biotechnology, Beijing, China), and a DAB kit was used to process the immunoreaction results. Three slices per brain region were analyzed in all experiments with data (cell counts) averaged per animal across slices.

### 2.5. BrdU Injection for Neurogenesis

To observe the neurogenesis in the dentate gyrus of the hippocampus, 5-bromodeoxyuridine (BrdU, 10 mg/ml, Sigma-Aldrich) labeling dissolved in 0.9% saline was used in rats. After tinnitus examination, rats were injected with 50 mg/kg BrdU at 9:00 a.m. Brains were collected for BrdU immunohistochemistry 12 h after the last injection of BrdU. For quantification of BrdU+ cells, every fifth section (thickness = 25 *μ*m) throughout the hippocampus was collected. The sections were washed with 0.01 M (pH = 7.2) PBS (3 min × 3) and 0.1% Triton X-100. The primary antibody was then added (Abcam, 1 : 500, mice anti-BrdU; Abcam 8955), and the tissue was incubated at 4°C overnight. Then, the secondary antibody (Goat Anti-Alexa Fluor 488, ab150113) was added at room temperature for 1 h. The sections were then washed with 0.01 M PBS (3 min × 3) and mounted on slides, coverslipped with Fluoromount G (Beckman Coulter), and stored at −20°C. Fluorescent images were captured using an Olympus confocal microscope. The total number of BrdU+ cells in the DG was extrapolated for the entire volume of the hippocampus.

## 3. Statistical Analysis

Statistical analysis was performed with SPSS (version 13.0). Results were expressed as the mean ± SEM. We used Fisher's exact test to examine the differences in latency between the groups and one-way analysis of variance (ANOVA) followed by the least significant difference (LSD) test to analyze histologically and MWM score differences. The ASR and PPI were calculated by the maximal amplitude of the spontaneous motor activity from the maximal amplitude of the startle response. Subsequently, the mean of the ASR and PPI response per stimulus and animal was calculated. PPI (%) = 1 − (ASR−PPI)/ASR *∗* 100%. Statistical analysis was carried out using the statistical software SPSS (Version 13.0). For each animal, ASR and PPI were analyzed in one- and/or two-way ANOVA with separate factors of test frequency, gap length, or bandwidth. Post hoc analysis was carried out using Tukey's HSD or independent contrasts corrected for multiple comparisons with the Bonferroni-Holm procedure. Significance levels are indicated in figures as ^*∗*^*p* < 0.05, ^*∗∗*^*p* < 0.01, and ^*∗∗∗*^*p* < 0.001.

## 4. Results

### 4.1. Tinnitus Examination by PPI

**Figure 1:**
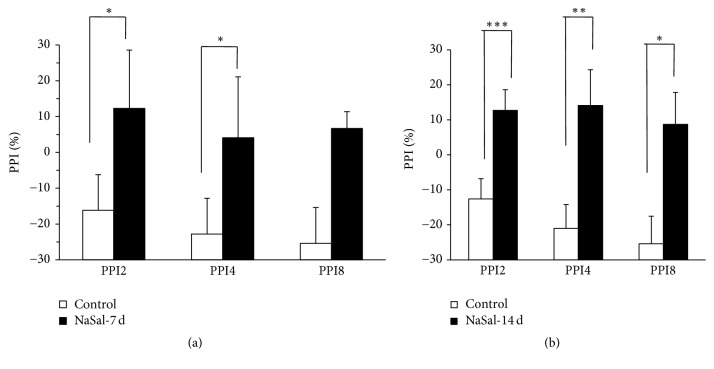
NaSal treatment induced tinnitus. (a) The PPI response measured in rats on the 7th day of NaSal treatment. (b) The PPI response measured in rats on the 14th day of NaSal treatment. *n* = 8–10; values are presented as mean ± SEM. Prepulse stimuli: PPI2, 75 dB SPL; PPI4, 80 dB SPL; and PPI8, 85 dB SPL (^*∗∗∗*^*p* < 0.001, ^*∗∗*^*p* < 0.01, and ^*∗*^*p* < 0.05, resp.).

After animals were treated for 7 consecutive days with NaSal or control injection (Figure 1(a)), PPI values for the different prepulse startle stimuli were measured and compared between groups: PPI2, −16.2  ±  −35.4 in the control group and 12.3 ± 16.3 in the NaSal group, *p* < 0.05; PPI4, −22.8  ±  −30.9 in the control group and 4.1 ± 17 in the NaSal group, *p* < 0.05; PPI8: −25.4  ±  −11.1 in the control group and 6.7 ± 4.7 in the NaSal group, *p* > 0.05; furthermore, no significant difference in the ASR was found between groups (*p* > 0.05). On the 14th day of treatment with NaSal or control injection (Figure 1(b)), studies indicated that more significant differences were found between groups: PPI2, −12.6  ±  −5.9 in the control group and 12.7 ± 5.4 in the NaSal group, *p* < 0.05; PPI4, −21  ±  −10.2 in the control group and 14.1 ± 6.8 in the NaSal group, *p* < 0.05; PPI8, −25.7  ±  −9.1 in the control group and 8.5 ± 7.9 in the NaSal group, *p* < 0.05; furthermore, no significant difference was found in the ASR between groups (*p* > 0.05). Because tinnitus occupied the gap space, rats treated with NaSal showed significantly lower PPI values than control rats.

### 4.2. NaSal Administration Inhibited the Learning and Memory of the Water Maze

Rats were trained in the MWM to test their spatial learning and memory abilities. After 7 consecutive days of NaSal treatment and the PPI test, the learning and memory abilities were tested in the water maze. During the learning phase, training was performed for 4 consecutive days. The results showed that the escape latency was progressively decreased during these 4 training days in all groups, but the rats treated with NaSal for 7 days (7 NaSal-treated rats) showed a decreased reduction in escape latency on the 4th day compared to the control rats (control group: 6.3 ± 1.4; 7 NaSal group: 11.8 ± 4.7, *p* < 0.05).

During the probe test, the 7-day NaSal-treated rats spent more time getting to the target quadrant on the 5th day compared to the control rats (control group: 8.3 ± 3.3; 7 NaSal group: 13.6 ± 9.1, *p* < 0.05), indicating that memory extinction was slower in the NaSal-treated rats than in the control rats.

**Figure 2:**
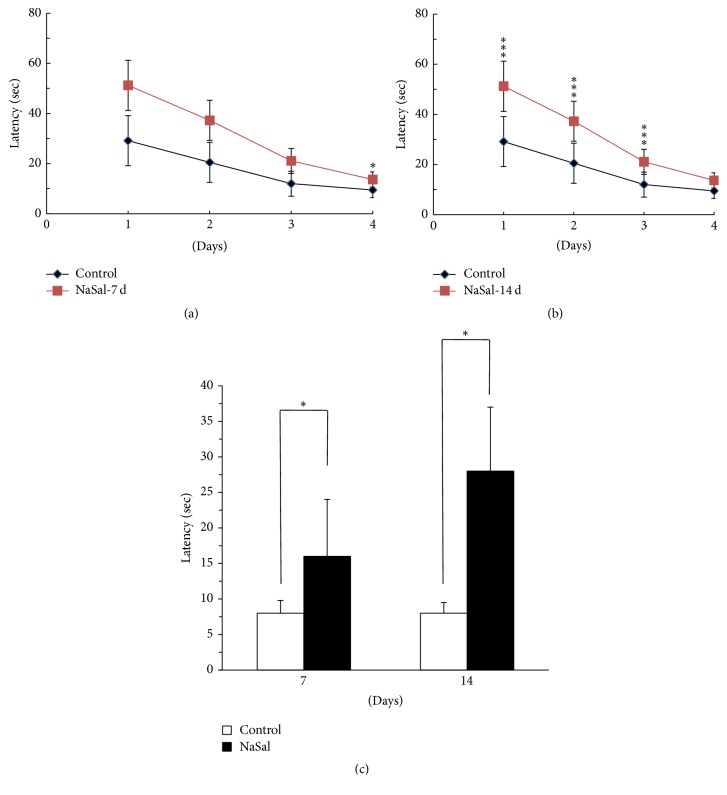
NaSal administration inhibits learning and memory of the water maze in rats. NaSal administration impaired the acquisition of spatial memory in the MWM as shown in (a) and (b) at 7 and 14 days, respectively. Also, the NaSal treatment for 7 and 14 days impaired the retrieval of the spatial memory in the MWM (c). *n* = 8–10, mean ± SEM (^*∗*^*p* < 0.05 and ^*∗∗∗*^*p* < 0.001).

After chronic (14 days) NaSal treatment and the PPI test, training was performed across 4 consecutive days during the learning phase. The results showed that the escape latency of every group showed a progressive decrease in the 4 training days, but the 14 NaSal-treated rats showed a smaller reduction in escape latency on the 4th day than the control rats (control group: 6.3 ± 1.4; 14 NaSal group: 11.8 ± 4.7, *p* < 0.05). During the probe test, the 14 NaSal-treated rats spent more time getting to the target quadrant on the 5th day than the control rats (control group: 8.3 ± 3.3; 14 NaSal group: 13.6 ± 9.1, *p* < 0.05), indicating that the memory extinction of the NaSal-treated rats was slower than that observed in the control rats (Figures 2(a) and 2(b)).

### 4.3. NaSal Administration Impaired the Expression of c-Fos during the Acquisition of Spatial Memory

**Figure 3:**
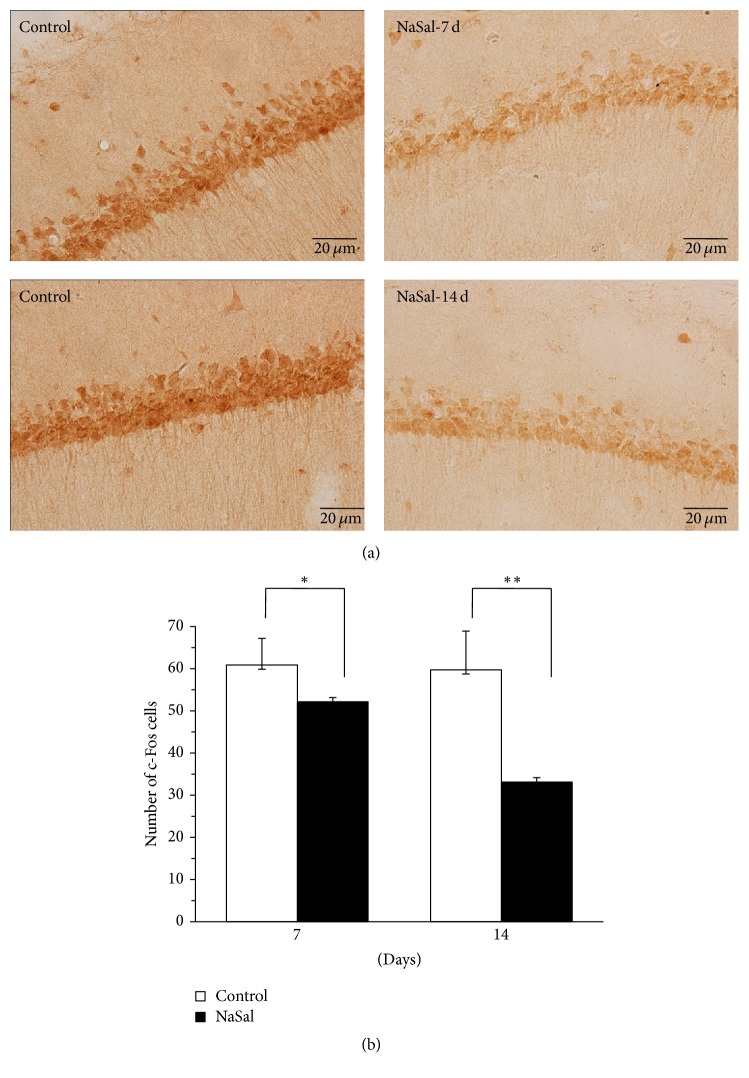
NaSal administration impaired the expression of c-Fos during the acquisition of spatial memory. (a) Representative immunohistochemical staining of c-Fos-positive cells in the hippocampal CA1 region from NaSal-treated and control rats. (b) Comparison of c-Fos expression during the acquisition of spatial memory in NaSal-treated and control rats. Independent-samples *t*-tests revealed significant effects on the 4th day of MWM training in rats given NaSal for 7 [*t* (17) = 2.334, *p* < 0.05] and 14 [*t* (17) = 5.912, *p* < 0.001] consecutive days. *n* = 8–10, mean ± SEM, ^*∗*^*p* < 0.05, ^*∗∗*^*p* < 0.001.

Spatial memory acquisition in the water maze is dependent on the activation of rapid gene expression (*fos* gene) in the hippocampus [14]. Therefore, we examined the expression of the c-Fos protein, which is dependent on neuronal activity [15]. The numbers of c-Fos-positive cells in the hippocampal CA1 region were compared between the treated and control groups after the water maze training on the 4th day using immunohistochemistry. Independent-samples *t*-tests revealed a significant decrease in the c-Fos expression on the 4th day after 7 days [*t* (17) = 2.334, *p* < 0.05] and 14 days of consistent NaSal administration [*t* (17) = 5.912, *p* < 0.001] (Figure 3(b)). The NaSal-treated animals (treated for 7 days and 14 days) showed a significant decrease in the c-Fos-positive cells compared with the control animals after MWM training. These results indicated that NaSal administration inhibited the induction of c-Fos expression in the CA1 of the hippocampus during acquisition of spatial memory in MWM training.

### 4.4. NaSal Administration Impaired the Expression of delta-FosB during Spatial Memory Retrieval

**Figure 4:**
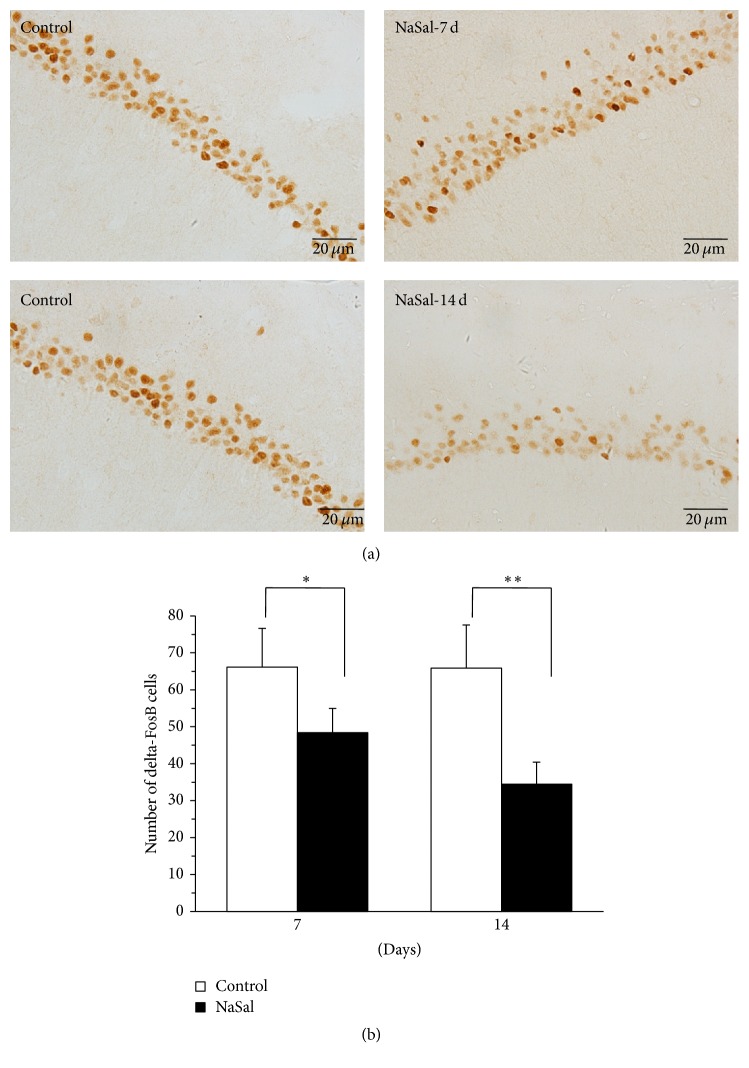
NaSal administration impaired the expression of delta-FosB during spatial memory retrieval. (a) Representative immunohistochemical staining of delta-FosB-positive cells in the hippocampal CA1 region from NaSal-treated and control rats. (b) Contrasting expression of delta-FosB during the retrieval of the spatial memory in NaSal-treated and control rats. Independent-samples *t*-tests revealed that NaSal treatment showed a significant decrease on the day of the probe test in the rats treated for 7 [*t* (15) = 3.521, *p* < 0.05] and 14 [*t* (15) = 5.859, *p* < 0.01] days. *n* = 8–10, mean ± SEM, ^*∗*^*p* < 0.05, ^*∗∗*^*p* < 0.001.

Memory retrieval requires hippocampal-dependent learning and memory, and ΔFosB plays a significant role [16]. However, to date, it is unknown whether ΔFosB expression is affected by NaSal exposure during memory retrieval in rats. As expected, water maze training induced an enhancement of ΔFosB protein in the CA1 of the hippocampus, and exposure to NaSal significantly inhibited expression of the ΔFosB protein in the CA1 of the hippocampus in the rats exposed to NaSal for 7 (*t* (15) = 3.521, *p* < 0.05) and 14 (*t* (15) = 5.859, *p* < 0.01) days. The NaSal-treated animals showed significantly decreased ΔFosB-positive cells compared with the control rats during retrieval (Figure 4). These results indicated that NaSal exposure inhibited the induction of ΔFosB in the CA1 of the hippocampus following training.

### 4.5. NaSal Administration Inhibited Neurogenesis in the DG of the Hippocampus

**Figure 5:**
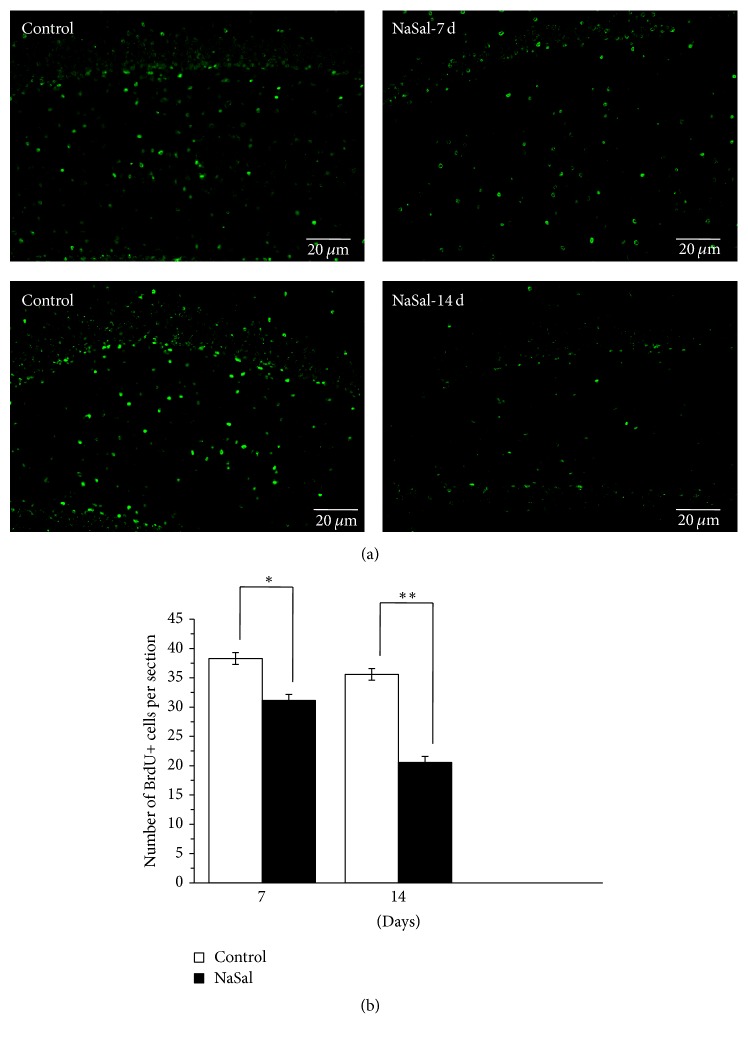
NaSal administration inhibited neurogenesis in the DG of the hippocampus. (a) Representative immunohistochemical staining of BrdU-positive cells in DG of the hippocampus in NaSal-treated and control rats. (b) Statistical analysis showed that consistent (7 days or 14 days) NaSal administration inhibited neurogenesis in DG of the hippocampus [7 days: *t* (14) = 2.374, *p* < 0.05; 14 days: (*t* (14) = 4.876, *p* < 0.01)]. *n* = 8–10, mean ± SEM, ^*∗∗*^*p* < 0.001.

BrdU staining, a marker of neurogenesis, was used to assess cell proliferation in the DG of both control and NaSal-treated rats. Consistent with previous studies, we detected strong BrdU staining in the hippocampus [17]. The BrdU+ cells were detected mainly in the subgranular zone of the DG. After MWM training, 7 and 14 days of NaSal treatment resulted in significant inhibition of BrdU staining in the DG [7 days: *t* (14) = 2.374, *p* < 0.05; 14 days: *t* (14) = 4.876, *p* < 0.01] (Figure 5). These results suggest that chronic NaSal administration inhibited neurogenesis.

## 5. Discussion

Various studies have indicated that environmental factors, including auditory and nonauditory factors, could be encoded into memories for places and events by the hippocampus. In adulthood, hippocampal neurogenesis plays a vital role in modulating memory function [3]. In the present study, we examined learning and memory abilities in a tinnitus model induced by NaSal administration, using the MW test for acquisition and retrieval of spatial memory. The results indicated that the abilities of acquisition and retrieval of spatial memory were impaired in the NaSal-treated animals. Moreover, NaSal treatment resulted in inhibition of c-Fos protein expression in the CA1 of the hippocampus after memory acquisition and delta-FosB protein expression in the CA1 after memory retrieval. In parallel, neurogenesis in the DG of the hippocampus was decreased by NaSal treatment.

NaSal is a well-known clinical drug that can induce reversible tinnitus and hearing loss [18]. Therefore, in animal studies, NaSal has been used to produce an animal model of tinnitus. Now, we established a tinnitus rat model using the long-term administration of sodium salicylate (200 mg/kg) twice daily for 7 or 14 consecutive days. Tinnitus behavior was confirmed by the PPI response, which was consistent with previous studies [19, 20]. Maladaptation of central system processing was thought to be responsible for tinnitus perception and generation. Previous fMRI and neurophysiological studies have indicated that the functions of the central auditory system and the prefrontal cortex, hippocampus, and emotional centers are disturbed in tinnitus, which are all brain regions considered to take part in mediating learning and memory. It has also been reported that structural abnormalities in the subgenual anterior cingulate cortex and the hippocampus are detected following salicylate administration [19, 20].

For the functional test, we examined the activation of gene expression in hippocampal neurons including the induction of c-Fos expression required for memory acquisition [14, 21]. The numbers of c-Fos-positive cells in the hippocampal CA1 region were decreased after consistent NaSal administration, indicating that NaSal administration inhibited the induction of c-Fos expression in the CA1 of the hippocampus during acquisition of spatial memory. As the expression of the c-Fos protein is tightly correlated to neuronal activity, our data suggest that, at least partially, the tinnitus may result in depressed neuronal activity in the CA1, leading to impairments of memory acquisition in the NaSal group. delta-FosB is a transcription factor within the Fos family that has been known to regulate synaptic plasticity in brain reward regions, such as the nucleus accumbens (NAc), prefrontal cortex, and ventral tegmental area [22, 23], and has a long half-life of 8 days in vivo after chronic stimuli [24]. Previous studies indicated that silencing the transcriptional activity of hippocampal delta-FosB impaired learning and memory across a battery of hippocampal-dependent memory tasks [16]. During our memory retrieval, the number of delta-FosB^+^ cells in the CA1 of the hippocampus was smaller in NaSal-treated rats than that observed in control rats. Considering that memory retrieval requires hippocampal-dependent learning and memory in which delta-FosB plays a significant role, we think that decreased delta-FosB level following tinnitus obviously contributes to the deteriorated memory retrieval. In general, the learning and memory abilities of NaSal-treated rats were shown to be impaired in MWM test compared to the control group, indicating that the network was disturbed with the NaSal administration. Taken together, these findings indicate that salicylate affects nonauditory regions of the CNS.

Additionally, neurogenesis has been implicated in learning and memory. Recently, it has been shown that neurogenesis in the adult hippocampus regulates hippocampus-dependent memories [25]. Previous studies have suggested that increasing hippocampal neurogenesis could accelerate forgetting, whereas inhibiting hippocampal neurogenesis could enhance the existing hippocampus-dependent memories [26]. In our studies, we first showed that neurogenesis of the DG field was impaired in NaSal-treated rats following water maze training during retrieval of the memory. It was previously reported that newborn neurons expressing c-Fos exert a long-term effect on DG function related to learning and memory [1]. Studies have indicated that tinnitus could arise from damage at any level of the auditory and nonauditory pathways, which could be induced by different factors leading to alterations in neuroplasticity in the auditory and nonauditory systems [27].

In conclusion, the results of this study support the hypothesis that tinnitus induced by NaSal affected the hippocampal system leading to memory dysfunction.
